# A Comprehensive Workplace Exercise Intervention to Reduce Musculoskeletal Pain and Improve Functional Capacity in Office Workers: A Randomized Controlled Study

**DOI:** 10.3390/healthcare12090915

**Published:** 2024-04-28

**Authors:** Konstantina Karatrantou, Vassilis Gerodimos

**Affiliations:** Department of Physical Education and Sports Science, University of Thessaly, 42100 Trikala, Greece; bgerom@uth.gr

**Keywords:** flexibility, balance, strength, occupational wellness, muscle disorder, work efficiency, work absenteeism

## Abstract

The high levels of musculoskeletal pain, in conjunction with the low levels of functional capacity, may negatively affect workers’ health, efficiency, and productivity. This randomized controlled study investigated the efficacy of a 6-month comprehensive workplace exercise program on musculoskeletal pain and functional capacity in office workers. Seventy male and female office workers with musculoskeletal pain in any body area were randomly assigned to either an intervention (IG; *n* = 35) or a control group (CG; *n* = 35). The IG participated every working day (during working hours) in a 6-month supervised combined (flexibility, strength, and balance) exercise program (120 training sessions; five times/week) for the total body. The CG did not participate in any intervention. Musculoskeletal pains in nine body areas and functional capacity (flexibility, balance, and strength) of the lower and upper body were measured before and following the intervention. The IG significantly reduced duration and intensity of pain (43.1–70%; *p* = 0.000) as well as days of work absenteeism (84.6%; *p* = 0.000), while improving work capacity (87.1%; *p* = 0.000). Furthermore, the IG significantly increased cervical, handgrip, back, and leg maximal strength (10.3–27.1%; *p* = 0.000) and flexibility and balance (12.3–73.7%; *p* = 0.000). In CG, all musculoskeletal pain and functional capacity indices remained unchanged. In conclusion, this program may be effectively used to reduce musculoskeletal pain and improve functional capacity.

## 1. Introduction

According to the European Union Labor Force Survey [1], office workers represent a significant proportion (approximately 30–35%) of the total working population (202,804,400 million in 2022) in Europe. Office workers spend a lot of time in the sitting position (approximately 70–85% of their time at work sitting), have low levels of physical activity inside and outside the work environment, and often adopt unhealthy behaviors in their daily lives (i.e., smoking, alcohol, ‘bad’ eating habits) [2,3,4,5]. The adoption of a “sedentary and unhealthy lifestyle” in conjunction with the high work demands affects office workers’ health-related quality of life (physical, mental, and social wellness) as well as their efficiency and productivity [4,5,6,7]. One of the most common health-related problems in the office working population (prevalence range from 37.9 to 95.3%) is musculoskeletal pains and disorders in different body parts (higher incidence at the cervical spine, shoulder, and back) [3,8,9,10,11,12], with detrimental effects not only for the office worker himself/herself and the workplace but also for society and the economy in general [12,13].

There is evidence that musculoskeletal pains and disorders are associated with increased absenteeism, lost productivity, and increased health care, disability, and/or worker’s compensation costs across all the European Union member states [12,13,14]. It is estimated that the total cost of lost productivity attributable to musculoskeletal disorders among the European working population is approximately EUR 240 billion, or up to 2% of the gross domestic product [13]. For this reason, the protection of the human body, the prevention and rehabilitation of musculoskeletal pains and disorders, the adoption of basic ergonomic principles, and the maintenance of satisfactory functional capacity levels (flexibility, strength, and balance) in different body areas are of crucial importance for healthy and independent living [15,16,17,18,19]. 

Until today, several studies in the scientific literature have examined the efficacy of different workplace intervention programs (i.e., ergonomic intervention, different exercise interventions, physical therapy), inside or outside working hours, to reduce musculoskeletal pains and disorders and to improve different features of physical fitness, health, and productivity in office workers [15,19,20,21,22,23,24,25,26,27,28,29,30,31,32,33,34,35,36,37,38,39,40]. It should be mentioned that the vast majority of the aforementioned studies focused only on the cervical, shoulder, and/or lower and upper back [19,20,21,22,23,26,27,32,34,35,36,38,39,40], while there is limited information on other body parts such as the elbow, forearm, leg, wrist, etc. [25,33,41,42]. However, previous studies showed that body parts such as the wrist, forearm, elbow, and lower limbs (knee, hip) are also painful for office workers [3,8,9,24], leading to absenteeism and/or reduced capacity to work [3,24]. Additionally, previous studies that examined the efficacy of different workplace intervention programs reported conflicting results regarding musculoskeletal pain (either reduction or no effect) and functional capacity (either improvement or no effect) [15,19,20,21,22,23,24,25,26,27,28,29,30,31,32,33,34,35,36,37,38,39,40,41,42]. Different factors, such as the type of intervention, the training characteristics (i.e., frequency of training per week), and the training goals (flexibility and/or strength and/or balance), as well as the subject’s characteristics, may account for these equivocal findings among studies. The frequency of training is one of the most important training characteristics that could influence the efficiency of an exercise program, where greater training frequency per week can lead to greater responses [43]. Several previous studies designed and implemented workplace exercise interventions from one to three times per week, aiming to improve a single index of functional capacity (flexibility or strength). The Center for Disease Control and Prevention (CDC) recommends that workers should daily participate in physical activities for at least 30 to 40 min [6], including a combined exercise program (flexibility, balance, and strength) [44,45]. Furthermore, previous studies in the scientific literature focused mainly on the evaluation of pain and quality of life using specific questionnaires [15,19,20,23,26,28,29,33,34,36,37,38,41], whereas limited studies have used functional capacity tests (flexibility and/or balance and/or strength) [21,22,27,35]. Nevertheless, the combination of specific musculoskeletal pain questionnaires with selected functional tests (flexibility, balance, and/or strength tests) may offer a more complete picture regarding the functional capacity of the musculoskeletal system in different body parts [46]. 

Taking all the above into consideration, the main objective of this study was to design, implement, and evaluate the efficacy of a daily 6-month workplace (during working hours) comprehensive combined exercise program (flexibility, strength, and balance) on musculoskeletal pain reduction (nine body parts: cervical spine, shoulder, upper back, elbows, wrists/hands, low back, hips/thighs, knees, and ankle/feet), on flexibility, balance (static and dynamic), and on maximal strength (cervical, handgrip strength, back, and leg), using simple, convenient, and portable equipment, in office workers. An additional and important objective of this study was to evaluate the individualized changes (individual responses per participant) following the 6-month intervention program on the musculoskeletal pain profile and functional capacity indices. This is of central importance since the individual characteristics (i.e., initial musculoskeletal pain and functional capacity profile, age) of the participants are an important factor that could affect the efficiency of the intervention program [43] and the generalization of study results in the whole population.

It was hypothesized that the 6-month intervention program would reduce musculoskeletal pain levels (intensity and duration) and improve functional capacity indices in each participant in the intervention group. It was also hypothesized that the baseline musculoskeletal pain and functional capacity profile would influence the percentage change following the intervention program. Thus, it was hypothesized that the participants with lower baseline functional capacity levels and higher baseline levels of musculoskeletal pains would report a greater percentage change in these indices following the intervention program.

## 2. Materials and Methods

### 2.1. Participants

Initially, 86 office workers with at least 8 h per day in the office (males and females; aged 30–53 years old) from five workplaces expressed interest in participating in this research and were assessed for eligibility according to inclusion and exclusion criteria. The history of musculoskeletal pain (duration and intensity of pain) in 9 body areas (cervical spine, shoulder, upper back, elbows, wrists/hands, low back, hips/thighs, knees, ankle/feet) of the participants was assessed prior to the start of the study using the Nordic musculoskeletal questionnaire [47] (Table 1). The participants should: (a) have musculoskeletal pain during the last month, at least in one of the nine examined body parts (cervical spine, shoulder, upper back, elbows, wrists/hands, low back, hips/thighs, knees, ankle/feet); (b) not suffer from chronic musculoskeletal pain; (c) not present musculoskeletal pain or discomfort the day of the measurement; (d) not have musculoskeletal injury the last 6 months; and (e) not require physical therapy or medical appointment. Additional exclusion criteria were: (i) chronic diseases; (ii) use of any medication that could affect the results of the study; and (iii) participation in organized exercise programs during the last 6 months. Nevertheless, the participants of both the IG and CG participated 2–3 times per week in non-organized physical activities, such as the use of bicycles for daily transportation, the use of stairs instead of elevators, and active participation in household and outside home chores. Both the IG and CG maintained these non-organized physical activities throughout the 6-month period, as this was evaluated before and at the end of the intervention period using a specific health history and daily habits questionnaire of the American College of Sports Medicine—ACSM [44].

**Figure 1:**
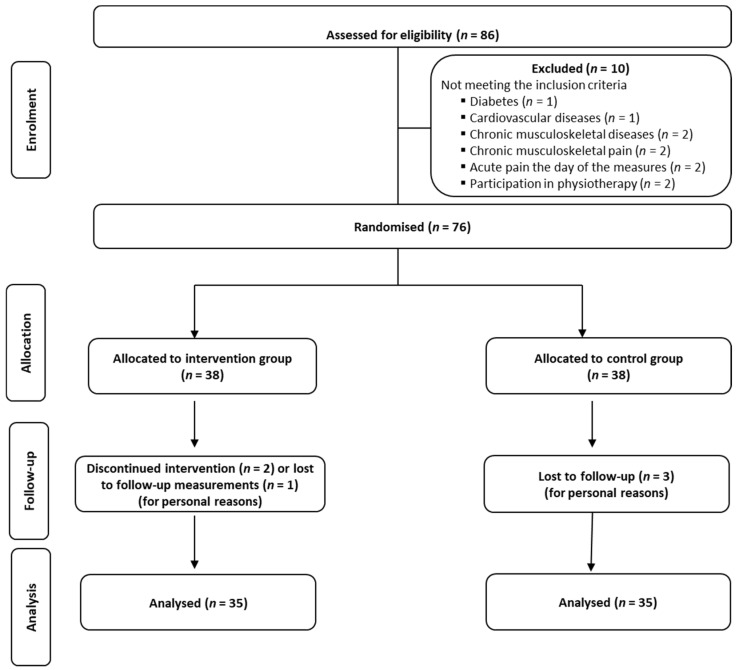
Flow chart of the progress through the phases of enrolment, intervention allocation, follow-up, and data analysis of a parallel randomized trial of two groups.

Of the 86 office workers who were assessed for eligibility, ten of them were excluded because they did not meet some of the above inclusion-exclusion criteria (Figure 1). Thus, 76 office workers volunteered to participate in the present study, and following baseline measurements, they were randomly assigned to either an intervention group (IG) or a control group (CG). Three participants from each group dropped out of the study (Figure 1), and therefore, the final number of participants was 70 (35 participants per group; Table 1).

The demographic, anthropometric, and working characteristics of the participants, which were assessed prior to the start of the study using the ACSM health history questionnaire [44], are analytically presented in Table 1. Before the start of the study, the participants were informed about the training and testing procedures and signed an informed consent form, and the Ethics Committee of the University of Thessaly in Greece approved the implementation of the present study.

### 2.2. Study Design—Procedure

**Figure 2:**
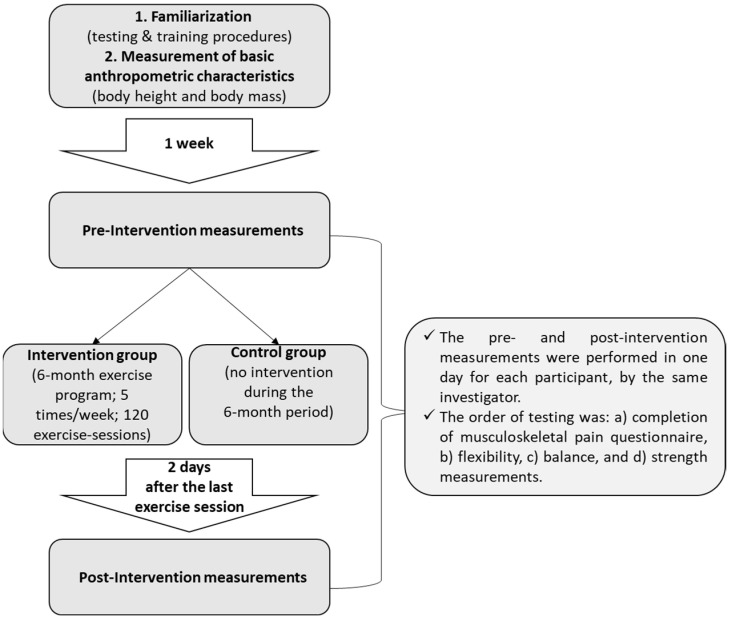
Study design—procedure.

The efficacy of the 6-month comprehensive workplace combined exercise program on musculoskeletal pain and functional capacity in office workers was investigated using a parallel randomized controlled design (Figure 1; Supplementary Table S1). A computer-generated list of random numbers was used for the allocation of the participants in one of the two groups. The main investigator was blinded for the allocated intervention during the entire period of data collection. During the study, participants were requested not to discuss their intervention with the main investigator. The total design and procedure of the study are analytically presented in Figure 2.

### 2.3. Intervention

Τhe 16-item internationally endorsed Consensus on Exercise Reporting Template (CERT) [48] was used to describe the exercise program of this study (Supplementary Table S2). The 16-item CERT checklist, which has been designed and developed by an international panel of exercise experts to improve reporting of essential components of exercise intervention programs, contains 7 sections/domains: what (materials); who (provider); how (delivery); where (location); when and how much (dosage); tailoring (what, how); and how well (compliance/planned and actual) [48].

The ΙG participated, every working day (5 days/week), in a 6-month supervised combined chair-based exercise program (120 training sessions; 25–38 min/day) that was implemented, in small groups (4–5 participants per group), at the workplace setting (during paid working hours) of the participants. The rationale for the selection of the appropriate intervention characteristics (total duration of the intervention: 6 months, frequency: daily, and duration of the exercise program: 25–38 min/day) was based on the recommendations of the ACSM [44,45] and CDC [6] as well as the findings of previous study in the scientific literature [24], which reported beneficial effects.

Each training session consisted of a 3–5 min warm-up, a 19–32 min main part of the program, and a 3–5 min cool-down. The main part of the exercise program included chair-based seated and chair-assisted standing exercises to improve flexibility (static stretching and dynamic exercises; 3–5 times/week; 1–2 sets/exercise; 10–20 s or reps/set), balance (static and dynamic exercises; 2–3 times/week; 1–3 sets/exercise; 8–20 reps or 10–30 s 3–6 m) and strength (bodyweight exercises and exercises with auxiliary training means; 2–4 times/week; 1–3 sets/exercise; 8–15 repetitions maximum—RM; slow to moderate speed) in the total body (Table 2). The training load gradually increased during the 6-month intervention program, according to the recommendation of the ACSM [44,45]. The gradual increase (per month) of the training load characteristics during flexibility, balance, and strength exercise workouts throughout the 6-month intervention program is analytically presented in Table 3.

The basic exercise equipment of the program was a stable chair (without wheels and armrests) with a back for better lumbar spine support (not a softback). The height of the chair was adjusted depending on the height of the participants so that the feet could be flat on the floor with a knee angle of approximately 90°. In addition to the basic exercise equipment (chair), other portable auxiliary exercise equipment was: (1) a rhythmic gymnastic ball (diameter: 16.5 cm; weight: 320 g); (2) a pair of silicone hand therapy balls (length/ball: 5.6 cm; width/ball: 4.2 cm; resistance: medium); (3) a pair of hand grippers (length/hand gripper: 12 cm; width/hand gripper: 8 cm; handle: plastic; resistance: medium); and (4) an exercise band (length: 1.5 m; resistance: medium/light blue color and heavy/blue color).

Before the start of the study, all the participants had similar levels (beginner level, according to their baseline measurements, which were compared with prior indicative values and norms in the scientific literature) of flexibility, balance, and strength. The exercise programs that were selected and used in the present study were similar for all the participants and were based on their initial level. However, there was some degree of tailoring in the training load characteristics according to the level of each individual. In more detail, there was a small adjustment of the duration (approximately 4–5 s) or repetitions (approximately 3–4 repetitions) per exercise according to the individual level of the participants.

An exercise instructor (a member of our research team) with special expertise and previous experience in occupational wellness exercise programs supervised the intervention program at the five workplaces and was responsible for the safe and efficient design, implementation, and guidance of the program. In order to ensure and strengthen exercise adherence during the 6-month intervention program, we used the following: (a) attendance books (with participants’ presences and absences) and (b) motivation strategies (i.e., thinking positively, committing, and setting goals) [50]. The motivation strategies were built into the daily exercise session, where the participants carried out the following: (a) were thinking positively regarding their participation in the exercise program; (b) were committed to regularly following the exercise program per day; and (c) were setting their short-term and realistic personal goals regarding their participation in the exercise program (using a goal-setting card). It should be mentioned that no injuries or adverse effects were reported during the implementation of the 6-month workplace exercise program.

### 2.4. Measurements

Musculoskeletal pains, flexibility, balance, and strength indices were measured before as well as after the completion of the 6-month workplace intervention program (Table 4) using various internationally recognized, widely used, reliable, and valid tests for middle-aged and older individuals [47,51,52,53,54,55,56,57]. Because all measurements were taken inside the workplace setting, we chose tests that do not require special space for their implementation and are carried out without any equipment or with small portable equipment. The test–retest reliability of these tests has been previously examined in office employees, inside the workplace environment, by our previous study [3], reporting high absolute and relative reliability indices (ICC = 0.95–0.98; SEM = 0.76–1.22; SEM% = 2.6–4). Before testing, the participants performed a standardized 12-minute warm-up (5 min of chair-based aerobic dance and 7 min of static and dynamic stretching exercises).

Participants were instructed to carry out the following: (a) abstain from any caffeine, tobacco, or alcohol consumption for at least 24 h before testing; (b) avoid physical activity for 48 h before testing; and (c) have sufficient rest the night before the testing.

### 2.5. Statistical Analysis

All statistical analyses were performed using IBM SPSS Statistics v.26 software (IBM Corporation, Armonk, New York, NY, USA), and the results are presented as the means ± standard deviations. A statistical power analysis (software package GPower 3.0) before the initiation of the study indicated that a total number of 40 participants (20 participants in each group) would yield adequate power (>0.85) and a level of significance (<0.05). The final total sample of the present study was 70 office employees (35 participants in each group). The normality of the data was examined using the Kolmogorov-Smirnov test (all variables followed the normal distribution). Two-way analyses of variances (2-way ANOVAs) [2 groups (IG and CG) × 2-time points (pre and post-intervention)] with repeated measures on the “time-point” factor and Sidak pairwise comparisons were applied to locate the significantly different means within and between groups. Independent t-tests were used to compare the percentage change (from pre- to post-measurement) between the two groups (IG and CG). The magnitude of the difference between groups was examined using Cohen’s *d* effect sizes (ES). Finally, Pearson correlation tests were used to examine a possible association between the pre-training musculoskeletal pain and functional capacity values and the magnitude of percentage change (from pre- to post-measurement). The level of significance for all statistical analyses was set at *p* < 0.05.

## 3. Results

### 3.1. Musculoskeletal Pains

Repeated measures analyses of variance (ANOVAs) revealed significant “group × time” interaction effects on duration and intensity of musculoskeletal pain in the cervical spine, upper back, lower back, shoulder, elbow, wrist/hand, hip, knee, and ankle/foot (*p* = 0.000). IG significantly reduced (*p* = 0.000) the duration and intensity of pains (Table 5 and Table 6) following the 6-month workplace exercise program, while in CG, the duration and intensity of pains remained unchanged (*p* > 0.05). Comparisons between groups revealed that pre-training values for duration and intensity of pain were not different between groups in none of the nine body areas assessed (*p* > 0.05), while all post-training musculoskeletal pain values (duration and intensity of pain) were significantly lower in IG vs. CG (*p* = 0.000). Independent *t*-tests showed that the percentage changes (from baseline to final measurements) in duration and intensity of pain in the cervical spine, upper back, lower back, shoulder, elbow, wrist/hand, hip, knee, and ankle/foot were significantly greater in IG than in CG (*p* = 0.000).

**Figure 3:**
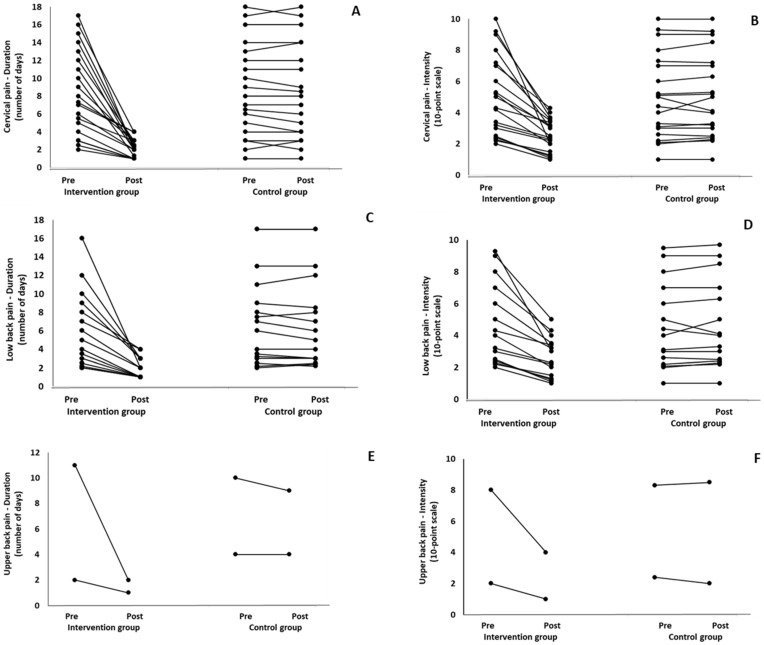
Individualized changes pre- and post-training in the duration (**A**–**C**) and intensity (**D**–**F**) of pain in the cervical spine, upper back, and lower back in the intervention and control groups.

**Figure 4:**
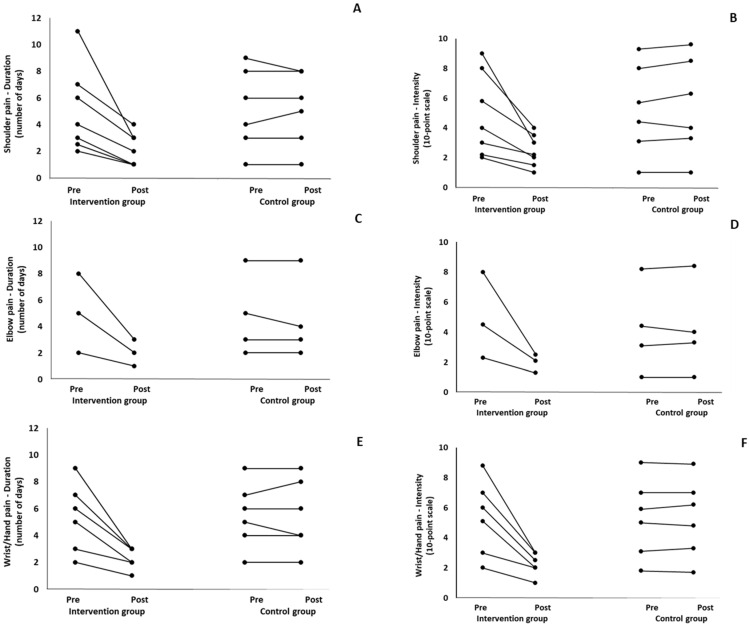
Individualized changes pre- and post-training in the duration (**A**–**C**) and intensity (**D**–**F**) of pain in the shoulder, elbow, and wrist/hand in the intervention and control groups.

**Figure 5:**
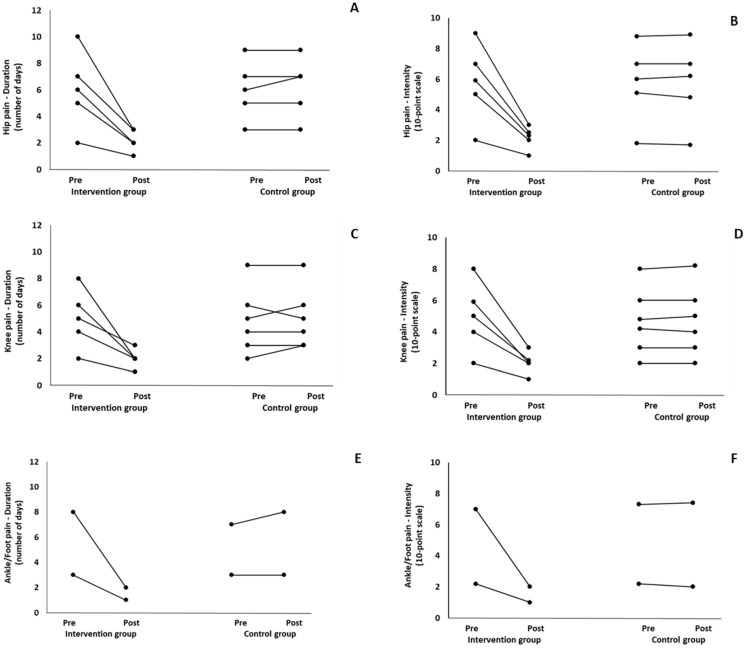
Individualized changes pre- and post-training in the duration (**A**–**C**) and intensity (**D**–**F**) of pain in the hip, knee, and foot/ankle in the intervention and control groups.

When the results were analyzed and presented individually, we found that all the participants in the IG with pain in the (a) cervical spine, upper back, and lower back (Figure 3); (b) shoulder, elbow, and wrist/hands (Figure 4); and (c) knee, hip, and ankle (Figure 5), following the intervention program, reduced the duration and intensity of pain in the suffering body parts. Also, Pearson correlation tests revealed significant positive associations between the baseline duration and intensity pain levels and the magnitude of the change at (a) the cervical spine, lower back, and upper back (*r* = 0.59–0.99; *p* = 0.01–0.001); (b) the hip, knee, and ankle (*r* = 0.72–0.98; *p* = 0.01–0.001); and (c) the shoulder, elbow, and wrist/hands (*r* = 0.66–0.99; *p* = 0.01–0.001). The participants with higher baseline duration and intensity pain levels reported a greater percentage change (reduction of duration and intensity of pain) following the 6-month intervention program. In the CG, when the results were examined individually, we observed conflicting results. Specifically, in some participants in CG, the duration and intensity of pains remained stable. While in others, these either increased or decreased following the 6 months.

Finally, ANOVA results also indicated significant “group × time” interaction effects (*p* = 0.000) on work absenteeism due to musculoskeletal pains as well as on the negative impact of musculoskeletal pains in participant’s daily activities. Specifically, IG following the intervention program significantly (*p* = 0.000) reduced the days of work absenteeism during the last month (pre-training: 6.5 ± 3.6 days and post-training: 1.0 ± 0.9 days; mean change: −84.6%; Cohen *d* = 2.4 large effect size). Additionally, IG following the intervention program significantly (*p* = 0.000) reduced the days of the negative impact of musculoskeletal pains in participant’s daily activities during the last month (pre-training: 11.6 ± 5 days and post-training: 1.5 ± 1.0 days; mean change: −87.1%; Cohen *d* = 3.4 large effect size). In CG, all the above variables remained unchanged.

### 3.2. Flexibility and Balance

#### 3.2.1. Flexibility (Sit-and-Reach Test and Back-Scratch Test)

Repeated measures analyses of variances indicated significant “group × time” interaction effects on the sit-and-reach test (*p* = 0.001) and on the back-scratch test (*p* = 0.001 for the right and left hands). In more detail, in the IG, post-training values on the sit-and-reach test (mean change: 22.9%; *p* = 0.001; Cohen *d* = 0.81 large effect size), as well as on the back-scratch test of the right hand (mean change: 60.9%; *p* = 0.001; Cohen *d* = 0.81 large effect size) and the left hand (mean change: 34.9%; *p* = 0.001; Cohen *d* = 0.80 large effect size), were significantly higher compared to the pre-training measurements. In CG, the sit-and-reach test and back-scratch test values remained unchanged after 6 months (*p* > 0.05) (Table 7). Comparisons between groups revealed that pre-training sit-and-reach test and back-scratch test values were not different between groups (*p* > 0.05), but all post-training values were significantly higher in IG vs. CG (*p* = 0.001). Independent *t*-tests showed that the percentage changes (from baseline to final measurements) in flexibility of the lower and upper limbs were significantly greater in IG than in CG (*p* = 0.000).

**Figure 6:**
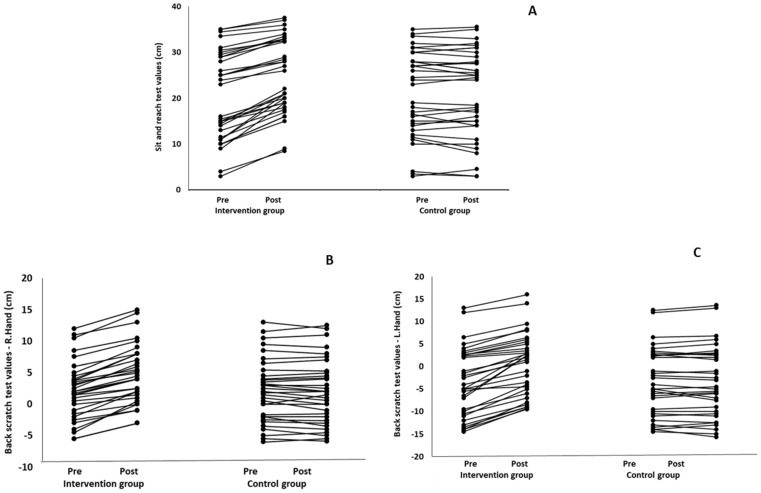
Individualized change (per participant) in the sit-and-reach (**A**) and back-scratch (right hand: (**B**) and left hand: (**C**)) tests from pre- to post-training for the intervention and control groups.

When the results were analyzed and presented individually (Figure 6), we observed that all the participants in the IG, following the intervention program, had increased sit-and-reach and back-scratch test values. The range of improvement was from 4.2 to 66.7% for the sit-and-reach test, from 10 to 100% for the back-scratch test on the right hand, and from 15 to 70% for the back-scratch test on the left hand, depending on the participant. Furthermore, in the IG, Pearson correlation analyses showed significant negative correlations between the pre-training flexibility values and the percentage change following the intervention program (*r* = −0.7 to −0.9; *p* = 0.000–0.001). The participants with lower pre-training sit-and-reach and back-scratch values reported a greater percentage change following the intervention program. On the other hand, in the CG, the individualized analyses indicated conflicting results (some participants reported no change, while others showed either a slight increase or decrease) following the 6 months.

#### 3.2.2. Balance (Single-Limb Stance Test and Timed Up-and-Go Test)

Repeated measures analyses of variances indicated significant “group × time” interaction effects on the single-limb stance test (*p* = 0.000 for the right and left legs) and on the timed up-and-go test (*p* = 0.000). In more detail, in the IG single-limb stance test, values were significantly higher at post-training compared to pre-training measurements for the right leg (mean change: 73.7%; *p* = 0.001; Cohen *d* = 3 large effect size) and the left leg (mean change: 70.7%; *p* = 0.001; Cohen *d* = 2.2 large effect size). Moreover, TUG (time in s values in the IG were significantly lower at post-training versus the pre-training measurements (mean change: −12.3%; *p* = 0.001; Cohen *d* = 1.1 large effect size). The CG single-limb stance test values and TUG values remained unchanged after 6 months (*p* > 0.05) (Table 7). Comparisons between groups revealed that all post-training single-limb stance test values were significantly greater in IG against CG, while TUG values were significantly lower in IG against CG (*p* < 0.001). In contrast, in the pre-training measurements, no significant differences were observed in static and dynamic balance between the two groups (*p* > 0.05). Independent *t*-tests showed that the percentage changes (from baseline to final measurements) in static and dynamic balance were significantly greater in IG than in CG (*p* = 0.000).

**Figure 7:**
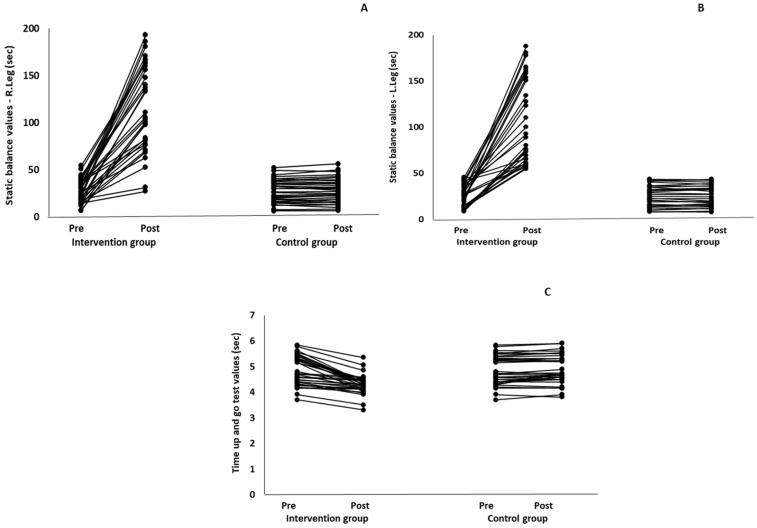
Individualized change (per participant) in the static-balance (right leg: (**A**), left leg: (**B**)) and timed up-and-go (**C**) tests from pre- to post-training for the intervention and control groups.

When the results were analyzed and presented individually (Figure 7), we observed that all the participants in the IG, following the intervention program, increased the single-limb stance test values (for the right and left legs) and reduced the time in the TUG test. The range of increase in the single-limb stance test was from 40.5 to 93.4% for the right leg and from 29.7 to 89.5% for the left leg, depending on the participant. Additionally, the range of reduction in time at the TUG test was from −1.5 to −28.6%, depending on the participant. It should be mentioned that, in the IG, Pearson correlation analyses showed significant negative correlations between the pre-training balance values (during the single-limb stance test and TUG) and the percentage change following the intervention program (*r* = −0.5 to −0.7; *p* = 0.001–0.01). The participants with lower pre-training levels reported a greater percentage change following the intervention program. On the other hand, in the CG, the individualized analyses indicated conflicting results (some participants reported no change; others showed either a slight increase or decrease) following the 6 months.

### 3.3. Strength

Significant “group × time” interaction effects were observed on maximal handgrip strength for right and left hands (*p* = 0.000), on cervical extension and flexion strength (*p* = 0.001), on back strength (*p* = 0.001), and on leg strength (*p* = 0.001) (Table 8). Post-training handgrip strength values for the right hand (mean change: 10.3%; *p* = 0.001; Cohen *d* = 0.82 large effect size) and for the left hand (mean change: 11.3%; *p* = 0.001; Cohen *d* = 0.80 large effect size), cervical strength values for extensor muscles (mean change: 20.3%; *p* = 0.001; Cohen *d* = 0.81 large effect size) and for flexor muscles (mean change: 20.8%; *p* = 0.001; Cohen *d* = 0.82 large effect size), back strength (mean change: 27.1%; *p* = 0.001; Cohen *d* = 0.82 large effect size) and leg strength (mean change: 25.1%; *p* = 0.001; Cohen *d* = 0.81 large effect size) values were significantly higher compared with pre-training values in IG. In CG, maximal strength values did not change throughout the study. Pairwise comparisons between groups revealed that at the post-training time point, maximal strength values were significantly greater in IG vs. CG (*p* = 0.001–0.000), whereas the pre-training maximal strength values were not different between groups. Independent *t*-tests showed that the percentage changes (from baseline to final measurements) in cervical, handgrip, trunk, and leg maximal strength were significantly greater in IG than in CG (*p* = 0.000).

**Figure 8:**
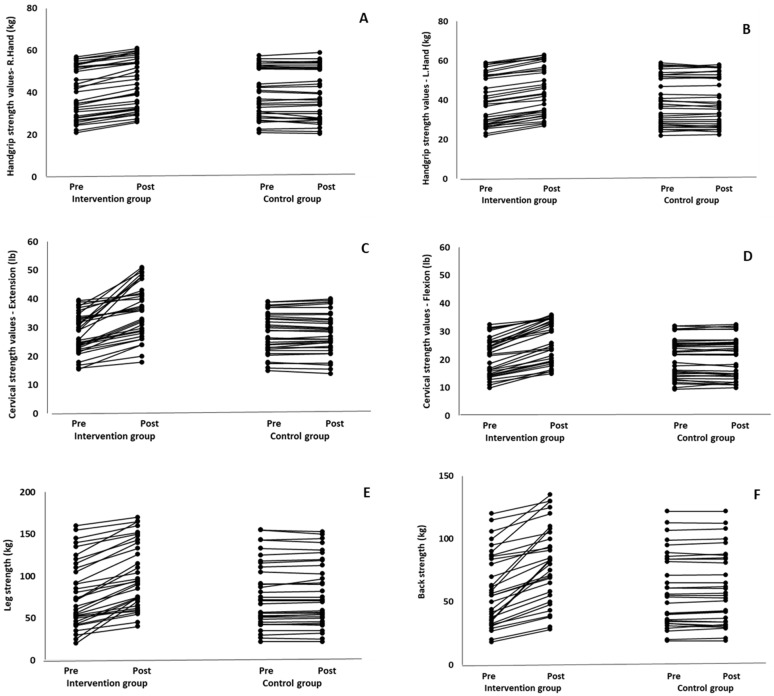
Individualized change (per participant) in the handgrip strength (right hand: (**A**), left hand: (**B**)), cervical strength (forward flexion: (**C**), extension: (**D**)), back strength (**E**), and leg strength (**F**) from pre- to post-training for the intervention and control groups.

When the results were analyzed and presented individually (Figure 8), we observed that all the participants in the IG, following the intervention program, increased all the evaluated strength parameters (handgrip, cervical, back, and leg maximal strength). The range of enhancement was from 3.3 to 24.1% for handgrip strength, from 6.3 to 37.5% for cervical flexion strength, from 2.5 to 45.8% for cervical extension strength, from 7.5 to 57.8% for back strength, and from 5.9 to 71.4% for leg strength, depending on the participant. It should be mentioned that, in the IG, the pre-training strength values were significantly associated (negative correlation) with the percentage change following the intervention program (*r* = −0.52 to −0.78; *p* = 0.000–0.01). Indeed, the participants with lower pre-training strength values reported a greater percentage change following the intervention program. On the other hand, in the CG, the individualized analyses indicated conflicting results (some participants reported no change; others showed either a slight increase or decrease) following the 6 months.

## 4. Discussion

This study designed, implemented, and evaluated a supervised daily 6-month workplace combined exercise program (flexibility, strength, and balance) for office workers with musculoskeletal pains that focused on nine areas of the human body (cervical spine, shoulder, upper back, elbows, wrists/hands, low back, hips/thighs, knees, and ankle/feet). Our results showed that the exercise program incorporated into the daily workplace routine demonstrated beneficial effects on functional capacity indices and musculoskeletal pain.

To our knowledge, this is the first study that designed, implemented, and evaluated a supervised daily 6-month workplace combined exercise program, offering a holistic functional capacity approach to the total body. The most important finding of the present study is the reduction of musculoskeletal pain characteristics in the intervention group following the 6-month workplace program. Before the study, the participants had musculoskeletal pains in various body parts that interfered with their daily activities in the workplace (11.6 ± 5 days the last month) and increased absenteeism from work (days of work absenteeism: 6.5 ± 3.6 days the last month). After the completion of the intervention program, there is a significant decrease in the duration (mean change: 52.9–70%, depending on the body area) and intensity (mean change: 43.1–63.3%, depending on the body area) of musculoskeletal pains, as well as in the work absenteeism due to musculoskeletal pains (84.6%) and the negative impact of pains in daily workplace activities (87.1%). The findings of the present study are in line with those of previous studies that examined the effects of specialized workplace exercise programs or physical therapy programs, reporting a significant reduction in musculoskeletal pain and work absenteeism [15,23,25,29,33,34,35,36,38,41]. Moreover, some other studies reported no significant effects of workplace interventions on workers’ musculoskeletal pains and disorders at different body parts [22,27,37]. The controversial—incompatible results among our study and previous studies that did not find beneficial effects on musculoskeletal pains may be attributed to different features, such as the type of intervention used (educational or counseling or physical therapy or exercise intervention), the training characteristics—specific goals of exercise interventions, the subjects’ characteristics, as well as the exercise supervision. For example, some of the aforementioned studies, which employed different intervention strategies, including individual counseling, educational or behavioral interventions regarding the use of sit-stand workstations, active rest, and/or ergonomics, demonstrated no change in musculoskeletal pain in office workers [37,60,61]. Based on the results of these studies [37,60,61], it seems that an alternative approach (specialized combined exercise programs emphasizing flexibility, balance, and strength) should be designed and implemented to provoke greater benefits for workers’ health, reducing musculoskeletal pain levels and improving all functional capacity indices, as observed in the present study. Our study and other studies [15,23,25,29,33,34,35,36,38,41] that implemented specialized workplace exercise programs reported significant beneficial effects on musculoskeletal pain characteristics. Furthermore, the training characteristics (e.g., training frequency per week) can affect the efficacy of the intervention program [43]. A previous study by Barros et al. [22] that implemented a 24-week (two times/week) workplace exercise program did not show significant effects on neck and shoulder pain intensity, independently of the level of adherence to the exercise program. On the other hand, in our study, we used a daily workplace exercise program that induced significant effects on pain elimination. As we mentioned earlier, an additional factor that could affect the efficiency of the intervention program is the individual characteristics of the participants (e.g., baseline functional capacity level, age) [43]. This notion has been strengthened by the results of the present study, where a significant association has been demonstrated between the baseline musculoskeletal pain-functional capacity levels and the magnitude of reductions following the intervention program. More specifically, office workers with greater duration and intensity of pains in the nine body parts showed greater reduction following the intervention program, while participants with lower baseline functional capacity levels showed greater adaptation following the intervention program. These findings are in line with the basic training principles [43], which mention that novice individuals with low levels of functional capacity have greater adaptations following an exercise program than trained individuals with higher baseline functional capacity levels.

Beyond the significant reductions of musculoskeletal pain characteristics, this study indicated that a 6-month combined exercise program, focused simultaneously on flexibility, balance, and strength of the total body, significantly improved all functional capacity indices (10.2–73.7%, depending on the measured parameter) in office workers. Our findings, regarding functional capacity indices, are in line with earlier studies that reported an improvement in various indices of functional capacity following different supervised flexibility and strength workplace exercise programs [27], whereas other studies reported no effect on some strength indices [21,22,35]. An important factor that seems to affect the efficacy of interventions is exercise adherence [22]. In more detail, Baros et al. [22] found greater adaptations in muscle strength and endurance of the shoulder joint in participants with high exercise adherence compared to participants with low exercise adherence. Moreover, previous studies that implemented exercise programs 2–3 times/week showed no significant effects on some indices of functional capacity (e.g., strength) [21,22], whereas the present study and previous study of Dalager et al. [27] that implemented the exercise program 5–6 times/week showed significant effects on all the evaluated strength parameters.

This study has some limitations that could affect the generalization of its findings. First of all, the results of this study are limited to middle-aged office workers (males and females) with musculoskeletal pains in different body parts and to the use of a 6-month workplace combined (flexibility, balance, and strength) chair-based exercise program. Upcoming studies could examine the efficacy of workplace intervention programs with different training characteristics and physical activities. Additionally, forthcoming studies could examine and compare the efficacy of similar exercise programs in target groups with different ages and working characteristics. An additional limitation of this study is the measurement of the parameters. Although we used a combination of musculoskeletal pain and functional capacity measurements, it would also be interesting to examine other biomechanical and psychological indicators that could affect musculoskeletal pain and disorders. The economic evaluation of the present study (e.g., cost-benefit analysis) could also be an important aspect of future research in occupational wellness and health scientific literature. Furthermore, in the present study, we did not consider the long-term effects of the intervention, examining the maintenance of positive changes and the continuation of the intervention program by the participants after 6 months. Another limitation of this study is the selection of time (during working hours) for the program implementation, the training frequency (every working day), and the duration of intervention per day (25–38 min). These factors could affect the proper functioning of the workplace, especially for workers with increased working obligations and limited time. Finally, the daily supervision of an intervention program by an exercise professional is a significant deterrent, especially for some workplaces that are not able to fund an exercise professional to implement the daily exercise sessions.

## 5. Conclusions

In conclusion, a daily 6-month workplace combined intervention program (during working hours) consisting of flexibility, strength, and balance exercises increases functional capacity indices and provokes significant reductions in musculoskeletal pain characteristics (duration and intensity of pains) in different body parts without causing adverse side effects or injuries. In our study, we decided to use an exercise program with low-cost equipment that may be effectively used in workplace settings without requiring a special space for its implementation. Furthermore, after the end of the 6-month intervention, the program may be implemented by the workers at the workplace or at home without supervision after appropriate education and instructions.

## Figures and Tables

**Table 1:** Demographic, anthropometric, musculoskeletal pain, and working characteristics in the IG and CG.

Variables	IG (*n* = 35)	CG (*n* = 35)
Demographic and Anthropometric characteristics		
Sex	21 ♀–14 ♂	20 ♀–15 ♂
Age (years)	43.9 ± 6.8	44.1 ± 5.9
Body height (m)	1.7 ± 0.1	1.7 ± 0.1
Body mass (kg)	79.9 ± 19.7	78.8 ± 19.6
Body mass index (kg/m^2^)	27.0 ± 5.7	27.2 ± 5.8
Musculoskeletal pain characteristics		
Incidence of pain per body part (%/number of participants)		
Cervical spine	57.1%/20	60.0%/21
Shoulder	20.0%/7	17.1%/6
Upper back	5.7%/2	5.7%/2
Elbow	8.6%/3	11.4%/4
Wrist/Hand	17.1%/6	17.1%/6
Low back	45.7%/16	42.9%/15
Hip/Thigh	14.3%/5	14.3%/5
Knee	14.3%/5	17.1%/6
Ankle/Foot	5.7%/2	5.7%/2
Duration of pain (days in the last month)		
Cervical spine	8.5 ± 4.8	8.5 ± 5.0
Shoulder	5.1 ± 3.2	5.2 ± 3.1
Upper back	6.5 ± 6.4	7.0 ± 4.2
Elbow	5.0 ± 3.0	4.8 ± 3.1
Wrist/Hand	5.3 ± 2.6	5.5 ± 2.4
Low back	6.6 ± 4.5	6.6 ± 4.4
Hip/Thigh	6.0 ± 2.9	6.0 ± 2.2
Knee	5.0 ± 2.2	4.8 ± 2.5
Ankle/Foot	5.3 ± 3.2	5.0 ± 2.8
Intensity of pain (score on a 10-point scale for the last month)		
Cervical spine	5.1 ± 2.6	5.0 ± 2.6
Shoulder	4.9 ± 2.8	5.0 ± 2.8
Upper back	5.0 ± 4.2	5.2 ± 4.3
Elbow	4.9 ± 2.9	4.7 ± 3.0
Wrist/Hand	5.4 ± 2.5	5.3 ± 2.6
Low back	4.9 ± 2.6	4.6 ± 2.7
Hip/Thigh	5.8 ± 2.6	5.7 ± 2.6
Knee	4.9 ± 2.2	5.1 ± 2.3
Ankle/Foot	4.7 ± 3.5	4.8 ± 3.6
Working characteristics		
Working experience (years)	18.5 ± 7.0	18.7 ± 6.4
Working hours/day (hours)	8.8 ± 0.3	8.7 ± 0.2
Working hours/week (hours)	44.0 ± 3.0	43.5 ± 2.5

**Table 2:** Flexibility, strength, and balance exercises of the intervention program [49].

Flexibility Exercises (Upper and Lower Body)			
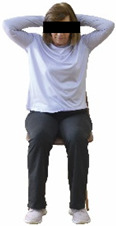	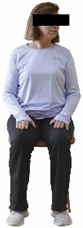	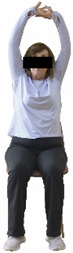	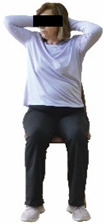
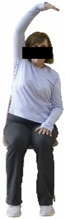	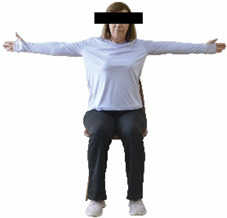	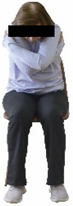	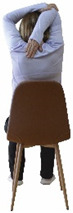
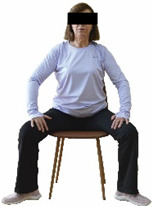	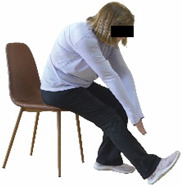	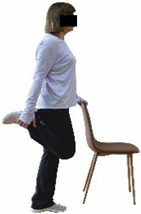	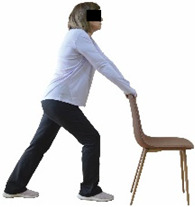
Strength exercises			
Cervical strength exercises			
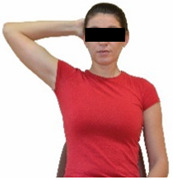	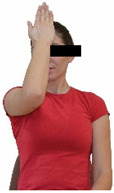	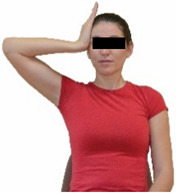	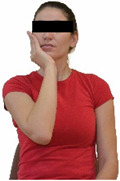
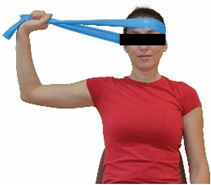		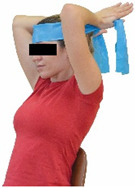	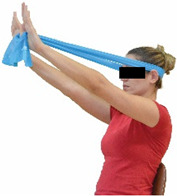
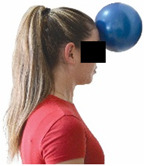 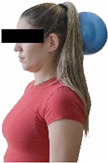 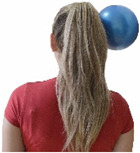			
Upper body strength exercises			
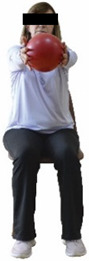	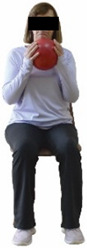	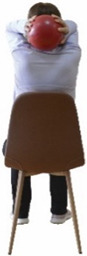	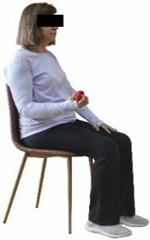
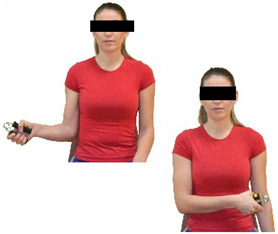	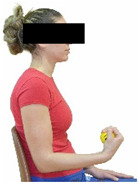	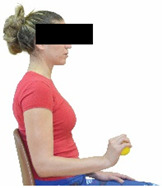	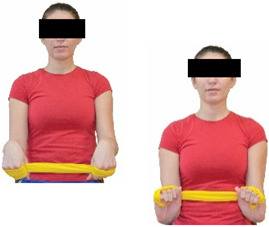
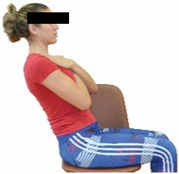	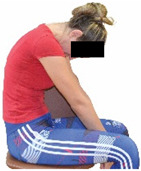	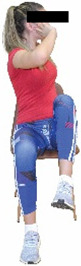	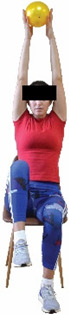
Lower body strength exercises			
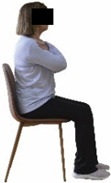	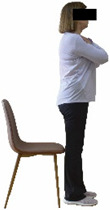	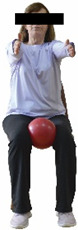	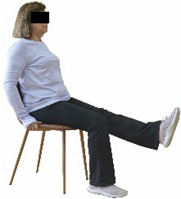
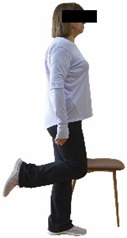 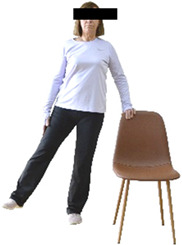 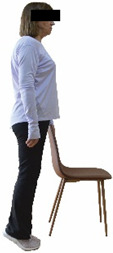			
Balance exercises			
Static exercises			
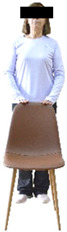	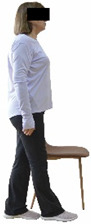	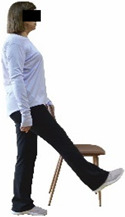	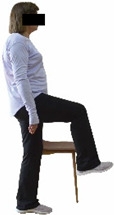
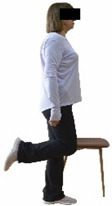	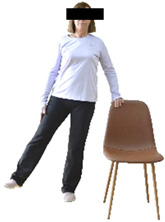	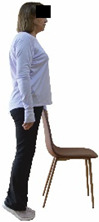	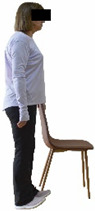
Dynamic exercises (by moving forward or backward)			
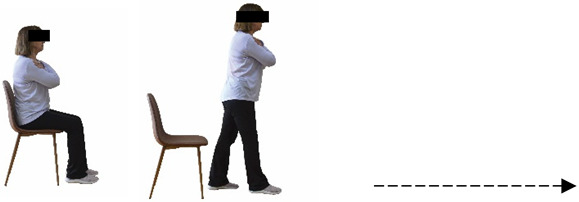		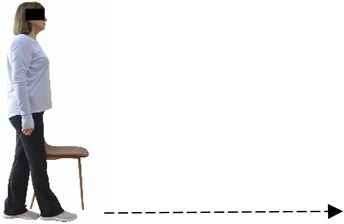	
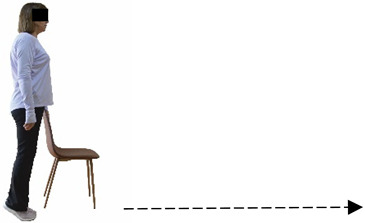		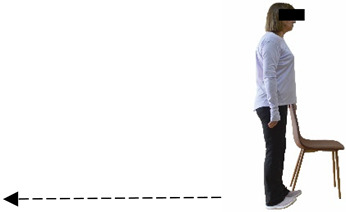	

**Table 3:** Training load progression during the 6-month intervention program.

	Months					
	1	2	3	4	5	6
Flexibility training						
Training frequency (times/week)	3	3–4	4	4–5	5	5
Sets	1	1–2	2	2	2	2
Duration (s) or Reps	10	10–15	15	15	15–20	20
Auxiliary exercise equipment	without exercise equipment			without exercise equipment/ with rhythmic gymnastic ball and exercise band		
Balance training						
Training frequency (times/week)	2	2	2–3	3	3	3
Sets	1–2	2	2	2–3	3	3
Reps or duration (s) or distance (m)/set	8–10 reps/ 10–15 s/3 m	10–12 reps/ 15 s/3–4 m	12 reps/ 15–20 s/4 m	12–15 reps/ 20 s/4–5 m	15 reps/ 20–25 s/5–6 m	15–20 reps/ 25–30 s/6 m
Auxiliary exercise equipment	without exercise equipment		hand therapy balls and rhythmic gymnastic ball		hand grippers and rhythmic gymnastic ball	
Strength training						
Training frequency (times/week)	2	2	2–3	3	3–4	4
Sets	1–2	2	2	2–3	3	3
Reps/set	8–10	10	10–12	12	12–15	15
Auxiliary exercise equipment	without exercise equipment/with hand therapy balls and rhythmic gymnastic ball		hand therapy balls, hand grippers, and rhythmic gymnastic ball		hand grippers, exercise band, and rhythmic gymnastic ball	

**Table 4:** Musculoskeletal pains, flexibility, balance, and strength measurements before and after the intervention program.

Measured Index	Test/Protocol	Equipment	Reliability/Validity
Musculoskeletal pain 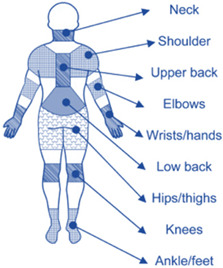	Duration and intensity of pain were evaluated in 9 body parts (cervical spine, shoulders, elbows, wrists/hands, upper back, lower back, hips, knees, and foot/ankle) during the last month. Days of absenteeism from work and the negative impact of musculoskeletal pains on participants’ daily activities were evaluated.	Nordic Musculoskeletal Questionnaire [47].	Test–retest reliability: Cronbach a = >0.95 [58]. Validity: >0.85 [58].
Lower back and hamstring flexibility 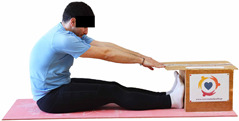	-The sit-and-reach test was used as previously described by [44]. -The best score (in cm) of three maximal trials (10 s rest/trial) was considered for analysis.	Flex-Tester box (Novel Products Inc., Rockton, IL, USA).	Intertrial reliability: ICC = 0.99 for men and women [59]. Test–retest reliability: ICC = 0.96–0.98 for men and women [3,59]. Inter-rater reliability: ICC = 0.98 for men and women [59].
Shoulder range of motion 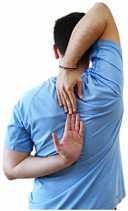	-The back-scratch test was performed as previously described by Corbin et al. [51]. -The best score (in cm) of three maximal trials (10 s rest/trial) at each hand was analyzed.	Measuring tape.	Test–retest reliability: ICC = >0.96 for middle-aged and older individuals [3,52]. Validity: ≥0.80 [52].
Static balance 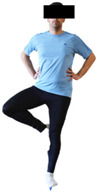	-The single-limb stance test with eyes opened was assessed on both legs, as previously described by Rinne et al. [53]. -The average (time in s) of the three trials at each leg was considered for analysis.	Stopwatch.	Test–retest reliability: ICC = >0.85 [53]–>0.95 [3]. Inter-rater reliability: ICC = 0.88–0.96 [53].
Dynamic balance 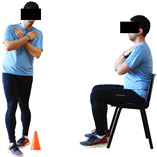	-The timed up-and-go test was used, as previously described by Rikli and Jones [52]. -The best time (in s) of three maximal trials (rest: 30 s/trial) was used for analysis.	Stable chair (without wheels and armrests), athletic cone, and stopwatch.	Test–retest reliability: ICC = >0.96 for middle-aged and older individuals [3,52]. Validity: ≥0.80 [52].
Cervical strength 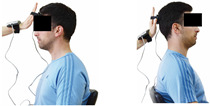	-The maximum isometric strength of cervical flexor and extensor muscles was assessed as previously described by Batatolis et al. [54]. -The best score (in lb) of three isometric contractions (1 min rest/trial) at each test was considered for analysis.	Hand-held dynamometer (JTech Commander PowerTrack II, Fabrication Enterprises Inc, NY, USA).	Test–retest reliability: ICC = >0.95 for middle-aged individuals [3].
Handgrip strength 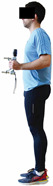	-Maximum isometric handgrip strength was assessed (the gold standard method for the evaluation of upper limb function) as previously described by Ruiz et al. [55] and Karatrantou and Gerodimos [3]. -The best score (in kg) of 3 isometric contractions (1 min rest/trial) at each hand was considered for analysis.	Portable hydraulic dynamometer (Jamar 5030J1, Horsham, PA, USA).	Test–retest reliability: ICC = >0.95 [3,55] for young and middle-aged individuals. Validity: ≥0.85 [55].
Back and leg strength 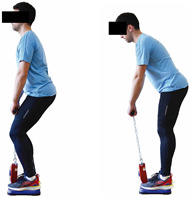	-Maximum back and leg strength were measured as previously described by Coldwells et al. [56] and Karatrantou and Gerodimos [3]. -A maximum of three trials were performed at each test (1 min rest/trial), and the best score (in kg) was considered for analysis.	Portable Takei back and leg dynamometer (Takei, Analogue dynamometer 5002, Takei Co., Niigata, Japan).	Test–retest reliability: ICC = >0.95 for middle-aged individuals [3].

**Table 5:** Intensity and duration of pain in the intervention and control groups pre- and post-training (mean ± SD).

	Intervention Group				Control Group			
Body Area	Duration of Pain (Days)		Intensity of Pain (Score, 10-Point Scale)		Duration of Pain (Days)		Intensity of Pain (Score, 10-Point Scale)	
	Pre	Post	Pre	Post	Pre	Post	Pre	Post
Cervical spine	8.5 ± 4.8	2.3 ± 1.0 *#	5.1 ± 2.6	2.6 ± 1.0 *#	8.5 ± 5.0	8.4 ± 5.1	5.0 ± 2.6	5.1 ± 2.6
Shoulder	5.1 ± 3.2	2.1 ± 1.2 *#	4.9 ± 2.8	2.5 ± 1.1 *#	5.2 ± 3.1	5.2 ± 2.8	5 ± 2.8	5.0 ± 2.8
Upper back	6.5 ± 6.4	1.5 ± 0.7 *#	5 ± 4.2	2.5 ± 2.1 *#	7 ± 4.2	6.5 ± 3.5	5.2 ± 4.3	5.3 ± 4.5
Elbow	5 ± 3	2 ± 1 *#	4.9 ± 2.9	1.9 ± 0.6 *#	4.8 ± 3.1	4.5 ± 3.1	4.7 ± 3.0	4.6 ± 3
Wrist/Hand	5.3 ± 2.6	2.3 ± 0.8 *#	5.4 ± 2.5	2.3 ± 0.7 *#	5.5 ± 2.4	5.5 ± 2.7	5.3 ± 2.6	5.3 ± 2.6
Low back	6.6 ± 4.5	2.1 ± 1.1 *#	4.9 ± 2.6	2.7 ± 1.3 *#	6.6 ± 4.4	6.4 ± 4.5	4.6 ± 2.7	4.7 ± 2.8
Hip/Thigh	6 ± 2.9	2.2 ± 0.8 *#	5.8 ± 2.6	2.2 ± 0.7 *#	6 ± 2.2	6.2 ± 2.3	5.7 ± 2.6	5.7 ± 2.7
Knee	5 ± 2.2	2 ± 0.7 *#	4.9 ± 2.2	2.0 ± 0.7 *#	4.8 ± 2.5	5 ± 2.3	5.1 ± 2.3	5.0 ± 2.2
Ankle/Foot	5.3 ± 3.2	1.5 ± 0.7 *#	4.7 ± 3.5	1.5 ± 0.7 *#	5 ± 2.8	5.5 ± 3.5	4.8 ± 3.6	4.7 ± 3.8

Where * *p* < 0.001 is the statistically significant difference between pre- and post-measurement in the intervention group and # *p* < 0.001 is the statistically significant difference between the intervention and control groups in the post-measurement.

**Table 6:** Mean percentage change, range of percentage change, and effect size from pre- to post-training in the intervention group.

	Mean % Change	Range of % Change	Effect Size (Cohen *d*)
Duration of pain			
Cervical spine	−68.3%	−42.9 to −88.2%	2.1 (large ES)
Shoulder	−56%	−42.9 to −72.7%	1.4 (large ES)
Upper back	−65.9%	−50 to −81.8%	1.4 (large ES)
Elbow	−57.5%	−50 to −62.5%	1.5 (large ES)
Wrist/Hand	−52.9%	−33.3 to −66.7%	1.8 (large ES)
Low back	−64.1%	−42.9 to −87.5%	1.6 (large ES)
Hip/Thigh	−60.8%	−50 to −70%	2.1 (large ES)
Knee	−56.3%	−50 to −75%	2.1 (large ES)
Ankle/Foot	−70%	−66.7 to −73.3%	1.9 (large ES)
Intensity of pain			
Cervical spine	−45.3%	−26.7 to −80%	1.4 (large ES)
Shoulder	−45%	−26.7 to −66.7%	1.2 (large ES)
Upper back	−50%	−48 to −52%	1.6 (large ES)
Elbow	−55.2%	−43.4 to −68.8%	1.7 (large ES)
Wrist/Hand	−54.3%	−33.3 to −65.9%	1.9 (large ES)
Low back	−43.1%	−20.9 to −67.7%	1.1 (large ES)
Hip/Thigh	−60.4%	−50 to −66.7%	2.2 (large ES)
Knee	−56.9%	−50 to −66.1%	2.0 (large ES)
Ankle/Foot	−63.3%	−54.6 to −72%	1.5 (large ES)

ES: effect size.

**Table 7:** Flexibility and balance values in the intervention and control groups pre- and post-training (mean ± SD).

Variables	Group	Pre-Training	Post-Training	Mean % Change
Flexibility				
Sit-and-reach test (cm)	IG	20.0 ± 5.1	24.5 ± 6 *#	+22.9 ^†^
	CG	21.3 ± 6.5	21.2 ± 6.2	−2.6
Back-scratch test—Right hand (cm)	IG	2 ± 4	4.8 ± 3 *#	+60.9 ^†^
	CG	1.8 ± 3.5	1.7 ± 4	−1.5
Back-scratch test—Left hand (cm)	IG	−2.9 ± 5	0.9 ± 4.5 *#	+34.9 ^†^
	CG	−2.9 ± 5.2	−2.9 ± 4.2	0.8
Balance				
Single-limb stance test—Right leg (s)	IG	28.6 ± 12.9	120.3 ± 48.2 *#	+73.7 ^†^
	CG	28.4 ± 12.7	28.7 ± 13.4	−0.4
Single-limb stance test—Left leg (s)	IG	32.5 ± 22.3	114.5 ± 51.2 *#	+70.7 ^†^
	CG	32.1 ± 22.5	32.1 ± 22.5	−0.3
Timed up-and-go test (s)	IG	4.8 ± 0.57	4.3± 0.37 *#	−12.3 ^†^
	CG	4.8 ± 0.58	4.9 ± 0.57	+0.98

Where * *p* < 0.001 is the statistically significant difference between the pre- and post-intervention programs in IG, # *p* < 0.001 is the statistically significant difference between IG and CG in the post-training measurement, and ^†^ *p* < 0.001 is the statistically significant difference between IG and CG in the percent change. CG: control group; IG: intervention group.

**Table 8:** Strength values in the intervention (IG) and control (CG) groups pre- and post-training (values are means ± SD).

Variables	Group	Pre-Training	Post-Training	Mean % Change
Handgrip strength (kg)				
Right hand	IG	38.5 ± 4.5	42.5 ± 5.5 *#	10.3 ± 4.3 ^†^
	CG	38.4 ± 4.7	38.3 ± 5.7	−0.5 ± 3.5
Left hand	IG	38.9 ± 5	43.4 ± 6 *#	11.3 ± 5.3 ^†^
	CG	38.6 ± 6	38.6 ± 6.5	−0.03 ± 2.6
Cervical strength (lb)				
Forward flexion	IG	20.9 ± 6	26.2 ± 7 *#	20.8 ± 8.4 ^†^
	CG	20.7 ± 6.8	20.6 ± 6.3	−0.9 ± 5.6
Extension	IG	28.1 ± 6.8	35.5 ± 9.1 *#	20.3 ± 11.5 ^†^
	CG	28.1 ± 6.9	28.3 ± 7.1	0.3 ± 3.2
Back strength (kg)	IG	56.7 ± 20	76.4 ± 28 *#	27.1 ± 13.7 ^†^
	CG	56.4 ± 22.5	56.9 ± 23.6	0.9 ± 3.7
Leg strength (kg)	IG	77 ± 24.1	99.7 ± 32.1 *#	25.1 ± 15.0 ^†^
	CG	76.8 ± 25	77.4 ± 33.5	0.8 ± 3.0

Where * *p* < 0.001 is the statistically significant difference between the pre- and post-intervention program in IG, # *p* < 0.001 is the statistically significant difference between IG and CG in the post-training measurement, and ^†^ *p* < 0.001 is the statistically significant difference between IG and CG in the percent change. CG: control group; IG: intervention group.

## Data Availability

Data are contained within the article.

## Supplementary Materials

The following supporting information can be downloaded at: https://www.mdpi.com/article/10.3390/healthcare12090915/s1, Table S1: CONSORT 2010 checklist of information to include when reporting a randomized trial [62]; Table S2: Consensus on Exercise Reporting Template (CERT) checklist.
